# Innate Immunity and Neuroinflammation

**DOI:** 10.1155/2013/342931

**Published:** 2013-06-15

**Authors:** Abhishek Shastri, Domenico Marco Bonifati, Uday Kishore

**Affiliations:** ^1^Centre for Infection, Immunity and Disease Mechanisms, Heinz Wolff Building, Brunel University, London UB8 3PH, UK; ^2^Unit of Neurology, Department of Neurological Disorders, Santa Chiara Hospital, Largo Medaglie d'oro 1, 38100 Trento, Italy

## Abstract

Inflammation of central nervous system (CNS) is usually associated with trauma and infection. Neuroinflammation occurs in close relation to trauma, infection, and neurodegenerative diseases. Low-level neuroinflammation is considered to have beneficial effects whereas chronic neuroinflammation can be harmful. Innate immune system consisting of pattern-recognition receptors, macrophages, and complement system plays a key role in CNS homeostasis following injury and infection. Here, we discuss how innate immune components can also contribute to neuroinflammation and neurodegeneration.

## 1. Introduction

Neuroinflammation is the mechanism of CNS inflammation that occurs in response to trauma, infections, and/or neurodegenerative diseases. In neuroinflammation, cellular and molecular immune components such as specialised macrophages (microglia), cytokines, complement, and pattern-recognition receptors are the contributing players. These proinflammatory mediators are either produced locally within the CNS or recruited from the peripheral system following disruption of the blood-brain barrier. This in turn leads to the activation of the glial cells, such as microglia and astrocytes. The effect of neuroinflammation is considered neuroprotective when the inflammatory activity is for a shorter period of time whereas chronic neuroinflammation is associated with harmful consequences for the CNS. 

Innate immunity is the first line of defence against the invading pathogens. Some of the components of first line of defence include epithelium (skin, gut, and lungs) that acts as a physical barrier and also produces several kinds of antimicrobial enzymes and peptides, namely, lysozyme, defensins, mucin, lectin [1]. Other components of innate immunity include the pattern-recognition receptors (PRRs) such as toll-like receptors (TLRs), nucleotide-binding, and oligomerisation domain, leucine-rich repeats containing (NOD)-like receptors (NLRs); and Scavenger receptors (SRs). Present on phagocytic and antigen-presenting cells, these receptors recognise not only exogenous pathogen-associated molecular pattern ^1^ (PAMP) but also endogenous modified molecules called damage-associated molecular pattern ^2^ (DAMP). The innate immune system launches inflammatory and regulatory responses via PRRs, phagocytes (macrophages), complement system, cytokines, and chemokines in order to counteract infection, injury, and maintenance of tissue homeostasis. Here, we discuss the role of innate immune players involved in neuroinflammation. 

## 2. Microglia

Microglial cells are the specialised resident macrophages of the CNS. The origin of these innate immune cells is debatable but it is now widely believed that they are of myeloid lineage [2]. In mice studies, it has been found that microglia originate from primitive (yolk sac) myeloid progenitors that migrate to CNS independent of definitive progenitors and circulation (i.e., bone marrow) [3]. These cells are found in brain, spinal cord, retina, and optic nerve. Their morphology differs from “conventional” macrophages by the presence of branch-like processes (ramified appearance). This is the shape they have when in “resting” state. In this state, these cells constantly monitor and survey their area [4]. The microglial cells in resting form have been shown to be involved in other functions such as neurogenesis [5], neuroprotection [6] and synaptic pruning [7], which has been found to be complement dependent [8]. Upon environmental stimulation/challenges, the microglia become “activated” and the morphology changes to an amoeboid appearance where they retract the ramifications [9]. Activation of microglia by TLRs and NLRs is considered to be “classical” form of microglial activation where innate immune responses include production of proinflammatory cytokines like tumour necrosis factor (TNF)-*α*, interleukin (IL)-1 and IL-6, and chemokines. Classical activation also leads to adaptive immune response by expressing major histocompatibility class II molecules and interaction with T cells [10]. TNF-*α* stimulation increases phagocytic activity of microglia [11], and deficiency of TNF receptors has been found to reduce microglial activation [12]. TNF-*α* is associated with activation of microglial cells involved in pathogenesis of neurodegenerative diseases like Alzheimer's disease (AD) [13] and Parkinson's disease (PD) [14]. IL-1 induces expression of TNF-*α* and IL-6 [15] and is implicated in neuroinflammatory processes in traumatic brain injury (TBI), AD, and PD [16]. Activated microglia have also been implicated in neurotransmission [17]. In order to regulate the immune responses, anti-inflammatory cytokines IL-10 and transforming growth factor beta are produced by microglia [18–20]. Microglia also produce inhibitor of nuclear factor *κβ*(NF-*κβ*), mitogen-activated protein kinase (MAPK) phosphatases, and suppressor of cytokine signalling proteins [21], which help in immune activation regulation. Glucocorticoids have also been considered to play a regulatory role for innate immunity in CNS by regulation of microglial TNF-*α* [22, 23] although there are debatable views to the same [24]. 

There are a variety of receptors expressed on microglia related to the different functions of these cells. Some of the receptors associated with innate immunity are listed in Table 1.

TLR 1–9 receptors are known to be expressed by microglial cells (discussed in detail later). NLR form complexes called inflammasomes (for a detailed review see [25]) that have been shown to activate and recruit microglia in response to amyloid-*β* (A*β*) [26] and prion peptide [27]. Some of the other innate immune receptors expressed on microglia surface are CD14, CD18, CD36, CD68, mannose, and lectin (Dendritic Cell-Specific Intercellular adhesion molecule-3-Grabbing nonintegrin or DC-SIGN) receptors. Complement receptors found on microglia are C3a, C5a, and C1q receptors [28].

## 3. Astrocytes

Astrocytes are specialised glial cells and the most abundant cells of the CNS. Morphologically, astrocytes are of two types: protoplasmic (found in grey matter) and fibrous (found in white matter). The basic astrocyte morphology resembles that of a star (with multiple processes). Protoplasmic astrocytes have undistinguishable dense processes while fibrous astrocytes have clearly distinguishable processes [29]. Astrocytes have conventionally been considered to be supporting cells to the neurons. However, recently they have been shown to play an active part in the modulation of neural activity [30], potentiation of synaptic transmission [31], sleep homeostasis [32], and even long-term memory formation [33]. Any insult to the CNS is associated with changes in the structure, morphology, and hypertrophy of astrocytes, followed by cytokine and C1q secretion, leading to scar formation, collectively termed as reactive astrogliosis [34].

Like microglia, astrocytes have been shown to express innate immune PRR like TLR, NLR, scavenger, complement, and mannose receptors [35]. They have also been shown to release cytokines like TNF, IL-6, IL-1, Interferon-*γ*, and chemokines when stimulated with lipopolysaccharide (LPS) [36, 37]. Reactive astrogliosis is associated with a number of CNS diseases such as AD [38, 39], PD, autism, and prion diseases [40, 41].

## 4. Toll-Like Receptors (TLR)

### 4.1. Structure and Signalling Pathway

**Figure 1:**
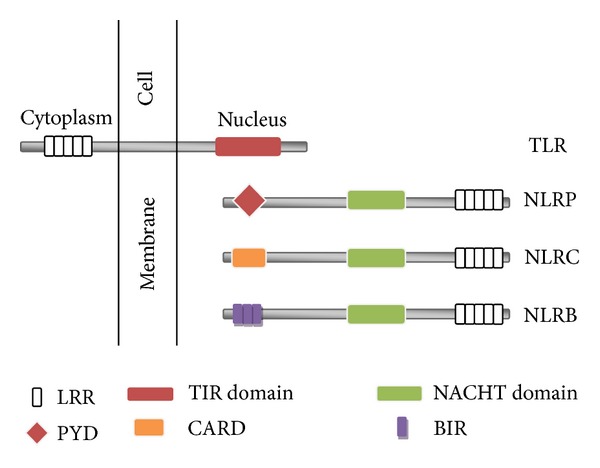
Schematic diagram showing structure of TLR and NLR family. TLR: toll-like receptor; NLRP: NOD-like receptor containing pyrin domain; NLRC: NOD-like receptor containing NLR-containing caspase activation and recruitment domain; NLRB: NOD-like receptor containing baculovirus inhibitor of apoptosis protein repeat domain; LRR: leucine-rich repeat; TIR: toll/il-1 receptor; PYD: pyrin domain; CARD: caspase activation and recruitment domain; BIR: baculovirus inhibitor of apoptosis protein repeat.   The figure shows the structure of a TLR containing a TIR domain present inside nucleus which is involved in signalling pathway and an LRR domain present in the cytoplasm which is involved in pathogen recognition. NLR are intracellular receptors containing a C-terminal LRR domain, a central NACHT domain, and a variable N-terminal domain which can be a PYD, a CARD, or a BIR domain.

TLRsare expressed on microglia, neurons, and astrocytes similar to dendritic cells, B cells, neutrophils, epithelia, and fibroblast [42]. TLR is a type 1 membrane protein containing an extracellular leucine-rich repeat (LRR) domain and a Toll/IL-1 receptor (TIR) domain in the cytoplasmic region (Figure 1). LRR domain is involved in specific pathogen recognition [43] and TIR domain is involved in the signalling pathway. TLRs are considered to exist as dimers and bind to various ligands [44, 45]. For example, TLR2 heterodimerises with TLR1 [46] and also with TLR6 [44] and recognises bacterial lipoproteins. Upon sensing ligands, recruitment of adaptor proteins takes place which is necessary for signal transduction [47]. The adaptor proteins are (i) myeloid differentiating factor 88 (MyD88); (ii) MyD88 adaptor-like protein (Mal); (iii) TIR domain-containing adaptor inducing interferon-*β* (TRIF); (iv) TRIF-related adaptor molecule; and (v) sterile-*α* and armadillo-motif-containing protein. These adaptor proteins are recruited by TIR domain leading to activation of NF-*κβ*. NF-*κβ* then induces production of proinflammatory cytokines such as TNF-*α*, IL-1*β*, and IL-6, and chemokines. All TLRs are activated by MyD88 except TLR3; instead MyD88 may be restricting TLR3 signalling [48]. Some of the other adaptors investigated in detail include major histocompatibility complex class II molecules [49], small heterodimer partner [50], and Dedicator of Cytokinesis 8 (DOCK8) [51].

It has recently been shown that oligomerisation of TLR4 with myeloid differentiation protein-2 by morphine causes neuroinflammation [52]. Necrotic neurons have been shown to activate microglia via MyD88 pathway leading to increased neuroinflammation [53]. In mouse models, both MyD88 and TRIF pathways have been implicated in regulation of IL-6 and IL-10 after cerebral ischaemia [54] as well as regulation of IL6, TNF-*α*, and IL-1*β* following intracerebral haemorrhage [55]. MyD88 pathway also plays an important role in CNS infection and consequent astrocyte activation [56]. MyD88 pathway may also be involved in PD [57] and optic nerve injury [58].

### 4.2. Ligands

Some of the exogenous and endogenous ligands of TLR are listed in Table 2 [59–62].

### 4.3. Response in CNS to Ligands of TLR

*In vivo *studies have shown that the administration of LPS (peripheral/intraperitoneal) leads to expression of genes coding for proinflammatory cytokines in the microglial cells [63, 64]. CD14 has been found to be required for LPS-induced endocytosis of TLR4 [65]. Injection of LPS directly into brain has been shown to produce an increased expression of genes of proinflammatory cytokines, chemokines, and complement proteins and receptors such as CD14 [66, 67]. Production of TNF by microglial cells upon LPS stimulation has been found to cause death of dopaminergic neurons [68]. TLR2 ligands stimulation of microglial and astrocytic cells leads to an increase in production of IL-6, chemokines, and IFN-*β* [69]. In mice studies, TLR9 ligand CpG has been found to be neuroprotective in cerebral ischaemia [70] while similar findings have been reported in TLR4 knockout mice [71]. TLR2 activation has been shown to be involved in neurogenesis [72] while TLR8 induces apoptosis of neurons [73]. TLR3 impairs plasticity and working memory [74] while TLR7 and TLR9 have been found to be associated with the development of mouse brain [75]. Interestingly, increased peripheral responses of TLR2, TLR4, TLR8, and TLR9 have been detected in psychosis [76] while TLR9 is associated with posttraumatic anxiety [77].

### 4.4. TLR Response to Pathogens

Pneumococcal infection leads to innate immune response in brain and this depends on TLR2 and TLR4 [78]. Deficiency of TLR2 causes an increased TNF gene expression in the brain [79]. TLRs have been found to be involved in pneumococcal infection in HIV-associated neurocognitive disorders [80]. TLR signalling is also associated with virulence of intracellular pathogens [81]. TLR2 and TLR9 initiate immune response against herpes simplex virus (HSV) [82] and also control HSV infection in the brain [83]. TLR3 is protective for the CNS in HSV1 infection [84]. In mice models, TLR3 in astrocytes may be protective in HSV2 infection [85] and has been reported to mediate entry of West Nile virus (WNV) into the CNS, causing encephalitis [86]. TLRs have also been implicated in CNS parasitic infections like toxoplasmosis,^3^ sleeping sickness,^4^ cerebral malaria,^5^ and neurocysticercosis^6^ [87]. TLR2 is associated with protection from cerebral malaria [88] and therapeutic targeting of TLRs has been shown to prevent experimental cerebral malaria [89, 90].

### 4.5. Neurodegenerative Diseases

In mouse model of AD, MyD88 has been found to prevent memory [91] and cognitive deficits [92] while another study found MyD88 deficiency to improve AD-related pathology [93]. TLR2 clears A*β* and delays cognitive decline, again in mouse model of disease [94]. TLR4 causes A*β*-induced microglial activation [95] and A*β*-induced neuronal apoptosis [96]. A loss-of-function mutation of TLR4 has been found to reduce microglial activation and increase A*β* deposits with increased cognitive deficits [97]. Intracranial injection of LPS (a TLR4 ligand) reduces A*β* levels in brain [98]. TLR9 may have a protective role in AD by improving cognitive functions [99], reducing A*β*-toxicity [100], and clearing A*β* [101]. In amyotrophic lateral sclerosis^7^ (ALS), MyD88 has been shown to activate microglia due to mutant SOD1 [102] and *in vitro* studies show enhanced microglial activation and neurotoxicity when stimulated with TLR2 and TLR4 ligands [103, 104]. MyD88 pathway may also be involved in PD [57] where *α*-synuclein directly activates microglia and alters expression of TLRs [105]. TLR signalling has been found to interfere with prion disease pathogenesis. Studies involving mice possessing mutant gene which prevents TLR4 signalling was found to have a shorter time for scrapie pathogenesis [106] while administration of TLR9 agonist in prion-infected mice leads to delayed onset of the disease [107]. However, MyD88 knockout mice (lacking TLR signalling) were found to develop prion disease similar to wild-type mice both in terms of time and severity [108].

## 5. NOD-Like Receptors

### 5.1. Structure

Like TLRs, NOD-like receptors (NLRs) also detect PAMPs and DAMPs. NLRs are intracellular receptors thereby monitoring intracellular environment. They consist of a central nucleotide-binding and oligomerisation (NACHT) domain and a C-terminal LRRs. Their N-terminal component may be variable based on which NLRs are further subdivided. It can be caspase activation and recruitment domain (CARD); a pyrin domain (PYD), or baculovirus inhibitor of apoptosis protein repeat (BIR) termed, respectively, as NLRC, NLRP, and NLRB [139]. Upon binding to agonists, NLR can lead to the activation of NF-*κβ* or MAPK signalling pathways and production of cytokines and chemokines. NLR binding to agonist also causes the activation of procaspase-1 leading to inflammasome formation; pyroptosis; autophagy; and IFN-1 signalling [140–145] (Figure 1) [141–145].

### 5.2. Inflammasomes

Inflammasomes are multiprotein complexes that activate caspase-1, which in turn leads to processing and secretion of proinflammatory cytokines such as IL-1*β* and IL-18. The members of NLR family that are capable of forming inflammasomes are PYD-containing NLRP1, NLRP3, NLRP6, and CARD-containing NLRC4 [146]. Inflammasome complex formation occurs when a ligand binds to NLR and thereby induces a conformational change, leading to ATP binding at NACHT domain which causes receptor oligomerisation and recruitment of other complex members [141]. Inflammasomes have been implicated in various diseases such as gout, pseudogout, contact dermatitis, allergic dermatitis, vitiligo, hydatidiform mole ^8^ [147], Muckle-Wells syndrome ^9^ [148], atherosclerosis, type 2 diabetes mellitus, obesity [149], metabolic syndrome ^10^ [150], acute myocardial infarction [151], coeliac disease, inflammatory bowel disease [152], asthma, pulmonary fibrosis [153], and viral [154] and bacterial infections [155].

### 5.3. Role in Neuroinflammation

NLRP3 inflammasome is involved in the innate immune response to A*β* [156] leading to AD pathology. In multiple sclerosis (MS), *NLRP3* knockout mice model of disease shows reduced demyelination [157], while another study shows NLRP3 involvement in migration of T-helper cells into CNS [158]. IFN-*β* therapy is effective in treating inflammasome-dependent disease in mouse models of MS [159]. NLRP1 has been found to be involved in TBI and neutralising its effect or formation was found to have beneficial effects [160]. Inflammasome complex inhibition has also been found to reduce inflammation and improve pathology in mouse models of stroke [161]. NLRP3 inflammasome contributes to brain injury in pneumococcal meningitis [162] and is associated with inflammation in Japanese encephalitis [163]. Both NLRP1 and NLRP3 are increased in postmortem alcoholic human brains and inhibition of these inflammasomes was found to be beneficial in reversing ethanol-mediated neuroinflammation [164].

## 6. Scavenger Receptors

### 6.1. Types

Scavenger receptors (SRs) are members of PRRs and are transmembrane glycoprotein PRRs [165]. SRs are expressed on macrophages, dendritic cells, microglia, and endothelial cells [111, 112]. Recently, SR expression on astrocytes has been reported [166]. The family of SRs include class A (macrophage receptors, MARCO), class B (CD36, SR-BI), CD68 and endothelial or LOX-1, CD163, and receptor for advanced glycation end products (RAGE) [167, 168]. Some of the ligands that SRs bind to are pathogen-specific: LPS, lipoteichoic acid, *Streptococcus pneumoniae*, *Staphylococcus aureus*, *Mycoplasma pneumoniae*, *Neisseriia meningitides*, *Escherichia coli* [169], apoptotic cells [170], and erythrocytes infected with *Plasmodium* [171–173]. SRs have been implicated in atherosclerosis [174], lung inflammation [175], cystic fibrosis [176], SLE [170], and AD [112]. 

### 6.2. Role in Neuroinflammation

Microglia express SR and thus bind to A*β* fibrils [177] which is associated with AD plaques [178]. Class A SR (SR-A) has also been shown to play an important role in cerebral injury due to ischemia. Mice deficient in SR-A showed reduced expression levels of TNF-*α* and IL-1*β* as well as decreased infarct size [179]. In experimental model of MS, SR-A knockout mice showed significantly reduced demyelination as well as reduced proinflammatory cytokines production [180]. However, deficiency of SR-A in AD mouse models was not found to impact amyloid plaque deposition or clearance [181]. *In vitro *studies have shown that astrocytes express SR-A and thus play a role in neuroinflammation [166]. Class B SR Type I (SR-BI) has been shown to be produced *in vivo *in AD brains [182] with increased expression being observed in cerebellum and cortex [183]. In mice studies, SR-BI has also been shown to impair perivascular macrophages leading to AD pathology such as increased amyloid deposition, cerebral amyloid angiopathy (deposition of A*β* in cerebral arteries), and memory deficits [184]. CD36 appears to be involved in neurovascular dysfunction due to A*β* [185] and promotes cerebral amyloid angiopathy leading to cognitive deficits [186]. RAGE is a receptor for A*β* and expressed on neurons, microglia, astrocytes, and endothelial cells [187]. RAGE signalling in microglia due to p38 MAPK signalling pathway leads to neuroinflammation and cognitive disturbances in AD [188] as well as synaptic [189] and neuronal [190] dysfunction. 

## 7. Complement

### 7.1. Three Activation Pathways of the Complement System

**Figure 2:**
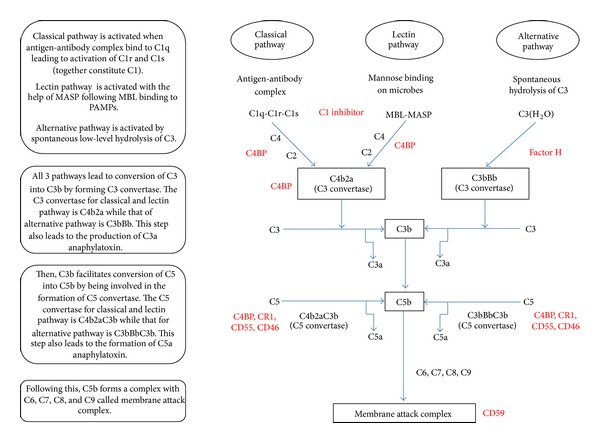
The complement system. Complement regulators are indicated in red. MBL: mannan-binding lectin; MASP: MBL-associated serine protease; C4BP: C4b-binding protein; CR1: complement receptor 1. The complement system consists of 3 initiating pathways: classical pathway, lectin pathway, and alternative pathway. The classical pathway is usually activated by antigen-antibody complexes, the lectin pathway is activated by microbes with MBL-MASP complex, and the alternative pathway is activated spontaneously by hydrolysis of C3 to C3(H_2_O). All 3 pathways lead to formation of C3 convertase, followed by C5 convertase, ultimately leading to formation of membrane attack complex. In this process, anaphylatoxins C3a and C5a are also released. The complement system is kept in check by a number of regulators.

The complement system comprises of more than 30 proteins in the serum as well as membrane-bound receptors and regulators. The complement system consists of 3 different initiating or activation pathways culminating into a final common lytic pathway, leading to the formation of membrane attack complex (MAC) (Figure 2). MAC are pores that penetrate cell membrane (lipid bilayers) of pathogens or abnormal cells, thereby causing their lysis. The three initiating pathways are called (i) classical pathway which is mostly antibody mediated (C1q being the first subcomponent) and is activated by C1 complex (C1q-C1r-C1s); (ii) alternative pathway (AP) which is activated spontaneously involving low-level hydrolysis of C3 to C3 (H_2_0); and (iii) lectin pathway where activation occurs through binding of a carbohydrate pattern present on microorganisms called mannan, with mannan-binding lectin (MBL) and Ficolins (ficolin-1, -2 and -3). They circulate in the serum in combination with zymogen serine proteases called MBL-associated serine proteases (MASPs) [191–196]. All the 3 pathways ultimately converge to lead to formation of C3 convertase. C3 convertases then cleaves C3 into C3a and C3b. This C3b binds to C3 convertase and leads to the formation of C5 convertase. This C5 convertase cleaves C5 into C5a and C5b. C3a and C5a are called anaphylatoxins and are chemoattractants. The C5b formed associates with C6, C7, C8, and C9 to form MAC [197]. The functions of the complement system include opsonisation of pathogens, direct lysis of foreign cells, chemotaxis and activation of leukocytes, and clearance of apoptotic cells. The complement system also interacts with TLRs [198] and plays a role in the regulation of humoral immunity [199]. The complement system is kept in check by regulators in order to prevent overactivation leading to damage to tissues and autoimmune diseases. The regulators can be grouped into fluid-phase: factor H (fH) and properdin for alternative pathway, C1 inhibitor and C4b-binding protein (C4BP) for classical and MBL pathway; host cell membrane-bound: CR1, CR2, CD55, CD46, CD59; cell surface-attached complement regulators: fH, factor H-like protein 1 (FHL-1), C4BP and clusterin [200, 201]. For certain ligands, factor H can also regulate C1q-mediated classical pathway [202–205].

### 7.2. Role in CNS Physiology

Complement is produced mainly in the liver and, over the years, it was thought that the brain was an immune-privileged organ due to the presence of blood-brain barrier. Now, it is well known that components of innate immunity like complement are present and even produced within the CNS. Neuronal cells [206–209], astrocytes [210, 211], and microglia [212–214] have been shown to produce complement and also express complement receptors. Role of complement in CNS is considered to be dual-neurotoxic and/or neuroprotective, depending on the level of its activation.

Complement has been shown to play a role in adult neurogenesis. Complement receptors C3aR and C5aR are expressed on neural stem cells and reduced neurogenesis is observed in the absence of C3aR signalling [215]. Another complement receptor CR2 has been found to be expressed in neural progenitor cells and also negatively regulates hippocampal neurogenesis [216]. An emerging area for complement involvement in CNS is in relation to synapse (reviewed in [217]). C1q, initiating component of classical pathway and widely expressed by postnatal neurons and immature astrocytes [218], mediates the elimination of synapse [219, 220]. C1q knockout mice show increased synaptic connectivity and spontaneous epilepsy [221]. Synapse remodelling by microglia involves CR3 [8]. *In vitro* studies show that C1q also promotes neuronal viability and survival [222]. *In vitro* and *in vivo* studies implicate a role for C3aR and C5aR in the development of cerebellum [223]. Many other *in vitro* and *in vivo* studies show neuroprotective functions for C3a and C5a that include protection against NMDA-induced apoptosis [224] and protection against glutamate-induced apoptosis [225] via MAPK-dependent inhibition of caspase 3 [226] as well as regulation of glutamate receptor subunit 2 [227]. 

### 7.3. Role in CNS Pathology

CNS can be infected by bacteria, virus, fungus, or protozoa. Deficiency of C3 is associated with increased susceptibility to meningococcal and pneumococcal infections [228]. Meningococcus binds to Factor H (fH), a negative regulator of alternative pathway, and evades host innate immune system [229, 230]. *Neisseria meningitidis* recruits host fH using protein mimicry [231]. Individuals with deficiency of properdin (positive regulator of alternative pathway) are susceptible to meningitis and individuals with combined properdin and MBL deficiency are at increased risk of infection with* Neisseria meningitidis *[232]. *Streptococcus pneumonia* infection of CNS is kept in check by complement system (mainly C1q and C3) [233]. C1q and C3 genetically deficient mice each showed considerably high bacterial titres in CNS as compared to wild-type mice. *Escherichia coli*, a cause for neonatal meningitis, crosses the blood-brain barrier by surviving in the serum where it binds to C4BP [234]. 

Viruses have also evolved mechanisms to evade complement system [235]. Gamma-herpesvirus encode for proteins that regulate and inhibit host C3-mediated resistance [236]. Complement controls antibody response in WNV infection [237] with lectin pathway activation being found to be protective in WNV infection [238]. C3 has been found to participate in seizure induction during viral encephalitis [239]. Increased MBL is seen in postmortem HIV encephalitis brains [240]. 

Fungal infection like cerebral aspergillosis leads to increased complement production seen in astrocytes, neurons, and oligodendrocytes, especially C1q production by infiltrating macrophages [241]. Some of the defence mechanisms developed by *Aspergillus fumigatus *to avoid complement include secreting fungal proteases [242] as well as production and recruitment of complement inhibitors [243]. In cerebral malaria, C1q and C5 levels have been found to be increased in mice studies [244] while another murine study also points to the requirement of MAC in the pathogenesis of cerebral malaria [245]. Infectious particles called prions cause CNS disorders like Creutzfeldt-Jakob disease and Bovine Spongiform Encephalopathy. These prion particles which activate classical complement pathway [246] are thought to bind to C1q and subsequently transported to the CNS [247]. C1q, C3b have been detected in postmortem brains of individuals with prion diseases [248], and MAC deposition was found to co-relate with prion disease severity [249].

Complement activation occurs in TBI and act as mediators of secondary brain injury [250, 251]. Following injury, levels of MAC corelate with blood-brain barrier (BBB) disruption [252]. In mice studies, absence of CD59 (a regulator of MAC formation) leads to increased neuropathology [253]. Postmortem studies on TBI brains show upregulation of C1q, C3b, and MAC [251]. Studies involving mice overexpressing complement inhibitor CR-related protein y (Crry) show reduced neurological impairment following TBI [254]. Hence, targeting complement activity in TBI may have therapeutic implications [255].

Cerebral ischemia can lead to the activation of the complement cascade leading to inflammation [256]. Systemic complement activity is also found to be enhanced in ischaemic stroke [257]. Complement system is implicated in ischemia reperfusion injury [258]. Ischaemic neurons have been found to produce C5a which causes apoptosis of neurons [259]. Better outcome is seen in individuals with low levels of MBL activity and mice lacking MBL [260]. Immunohistochemistry on brains of stroke patients shows C1q deposition while complement regulator CD59 was found to be absent [261]. Studies involving C5- [262] and C3-deficient mice [263] as well as C1 inhibition [264] have been successful in having beneficial effects in stroke therapy by targeting complement [256, 265].

A major role for complement is also seen in neurodegenerative diseases like AD. The neuropathology in AD includes loss of neurons, extracellular amyloid plaques, and intracellular neurofibrillary tangles consisting of abnormally phosphorylated tau protein [266]. A*β* activates complement [267], most notably via the classical pathway. Activated complement components C1q, C3d, and C4d have been detected in amyloid plaques [268, 269] by immunohistochemistry. C1q binds to A*β* [270, 271] and modulates phagocytosis of A*β* by microglia [272]. Upon exposure to A*β*, C1q is expressed in neurons (hippocampus) [273], and it has been found that inhibiting the binding of C1q to A*β* leads to protection of hippocampal cells [274]. In mouse models of AD, absence of C1q shows less neuropathology [275]. Complement regulators factor H, FHL-1, and C4BP have also been localised in amyloid plaques and fH and C4BP have been shown to bind A*βin vitro* [276–278]. These regulators could be involved in regulation of excessive complement activation. Another interesting feature is the presence of microglia expressing complement receptors found in close proximity to plaques. Microglia are found in and around plaques of AD brains [279] and are found to express C1q [280] and complement receptors C1qR, CR3, CR4, and C5aR, which help in the phagocytosis of A*β* [281, 282]. Complement activation is therefore also considered to be neuroprotective [266]. C3 deficiency in mouse model shows accelerated amyloid plaque deposition [283]. Furthermore, inhibition of complement was found to be associated with an increased formation of plaque and neurodegeneration [284]. Amyloid precursor protein transgenic mouse models of AD that lack the ability to activate classical pathway (APPQ^−/−^) (i.e., C1q^−/−^  phenotype) show less neuropathology as compared to APPQ^+/+^  mice. However, APPQ^−/−^  mice also show increased C3 levels, providing evidence for alternative pathway activation in AD [285]. In mice models, deficiency of sCrry increases tau pathology [286]. Genetic association of AD and complement involves complement genes *CR1* and *CLU *[287]. Micro-RNAs^11^ (miRNAs) −9, −125b, −146a, and −155 are found to be upregulated in AD and these miRNAs target gene encoding fH [288]. 

An emerging role for complement in MS has become evident recently [289]. C3d is localized along with microglia in MS tissues [290]. Priming of microglia in MS has been found to be C3-dependent and, in the same study, it was found that in animal model of MS, Crry-deficient mice show exacerbated and accelerated disease progression [291]. Serum factor H has been found to be a useful biomarker for MS [292]. Pathological studies of MS lesions have found presence of complement components C3d, C4b, C1q, and MAC on myelin sheath, surrounding vessel walls, microglia, and astrocytes [293–296].

There is evidence for neuroinflammation in PD as well [297] with the presence of reactive microglia and activated components of complement. Elevated mRNA levels of activated complement and markers of reactive microglia are also seen in PD [298]. Pathological studies show the presence of MAC components intracellularly on the characteristic Lewy Bodies [299, 300]. The cerebrospinal fluid levels of C3 and factor H have been observed to correlate with severity of PD [301]. An interesting study found a role for C1q in PD. Neuromelanin (NM) is a pigment that accumulates in dopaminergic neurons in normal aging process. In PD, these dopaminergic neurons are susceptible to degeneration [302] which is thought to be caused by activation of microglia by NM [303]. Furthermore, this NM pigment is found to be opsonised by C1q and phagocytosed by C1q-positive microglia [304]. 

Huntington's disease (HD) is another neurodegenerative disorder and a genetic cause of dementia. It is inherited as an autosomal-dominant trait characterised by abnormal (at least 36) CAG repeats on the coding sequence of *huntingtin* gene [305]. Neuropathological studies in HD brains show presence of complement components C1q, C4, and C3 along with upregulation of complement regulators C1 inhibitor, clusterin, CD59, and CD55. In this study, microglial expression of higher levels of C3 and C9 was also observed [306]. 

There has been increasing evidence for involvement of complement in schizophrenia. Schizophrenia is a psychiatric illness characterised by thought insertion, thought withdrawal, hallucinations, delusions, and negative symptoms such as apathy, speech problems, and slow cognition. There is an increase in serum levels of classical pathway complement proteins such as C1q, C1, C3, and C4; increased total complement activity (CH_50_), CR1 levels; and decreased C4BP levels [307–309]. The alternative pathway is also involved with increased factor B levels and increased activity in serum [310]. MBL pathway shows increased activity as well (increased MBL and MASP-2 levels) [311, 312]. Genetic studies have shown *C1QB* gene polymorphism, *CSMD1* and *CSMD2 *(code for complement regulatory proteins)*, C3, MBL2, *and *MASP2* gene association [313–316]. 

## 8. Conclusion

A role for innate immunity in inflammation of CNS is being increasingly evidenced. Cells of the CNS such as neurons, astrocytes, and microglia along with pattern recognition receptors, cytokines, chemokines, complement, peripheral immune cells, and signal pathways form the basis for neuroinflammation. Local synthesis of a number of innate immune humoral factors within CNS offers an opportunity for therapeutic intervention. Furthermore, excessive activation of immune system is thought to be destructive to tissues whereas, simultaneously, it opens up possibilities to harness this activation in a controlled manner to obtain desired therapeutic or preventive strategies in CNS diseases. A detailed understanding of the processes and mechanisms involved in the etiopathogenesis of CNS diseases as well as normal functioning of CNS immunity is essential and can pave the way for reducing excessive neuroinflammation and its effects. Modulation of cellular processes, phenotypes, and functions looks increasingly likely to be a way forward in combating CNS disorders.

## Figures and Tables

**Table 1:** Innate immune receptors on microglia.

Receptor	Functions/comments	References
TLR	Pattern-recognition receptors that respond to self (DAMPs) and nonself (PAMPs) activators. Microglia are known to express TLR1-9. TLRs are implicated in neuroinflammation in response to bacterial and viral infections, Alzheimer's disease, prion diseases, and amyotrophic lateral sclerosis.	[59, 69]

NLR	Cytoplasmic pattern-recognition receptors. Microglia are known to express NOD2 in response to CNS infection and NALP3 inflammasome in Alzheimer's disease.	[109, 110]

Scavenger	Another group of pattern-recognition receptors. The receptors expressed on microglia are Class A, CD36, and RAGE.	[111, 112]

RLR	RIG-I is a pattern-recognition receptor that is expressed by microglia in response to viral infections.	[110, 113]

Complement	Complement receptors expressed include CR1, CR3 and CR4. These receptors bind complement proteins and activate complement pathway which is considered to be both beneficial and detrimental depending on the level of activation.	[114]

Cytokines	Some of the cytokine receptors expressed in microglia are IL-1R, TNFR (responsible for proinflammatory actions of cytokines IL-1 and TNF-*α* resp.), IL-10R, TGFR (responsible for the anti-inflammatory cytokines IL-10 and TGF-*β*), and CCR1-5 responsible for actions of chemokines. These are expressed and produced in neuroinflammation.	[115, 116]

TLR: toll-like receptor; DAMP: damage-associated molecular pattern; PAMP: pattern-associated molecular pattern; NLR: NOD-like receptors; NOD: nucleotide-binding and oligomerisation domain; RLR: RIG-like receptors; RIG: retinoic acid-inducible gene; CR: complement receptor; IL: interleukin; TNF: tumour necrosis factor; TGF: transforming growth factor.

**Table 2:** Exogenous and endogenous ligands of toll-like receptors.

Ligand	TLR	Implications/comments	References
Lipopolysaccharide	TLR4	Recognition of Gram (−) bacteria	[117]
Triacylated lipopeptides	TLR1 and TLR2	Recognition of Gram (−) bacteria and mycobacteria	[118]
Diacylated lipopeptides	TLR2 and TLR6	Recognition of Gram (+) bacteria and mycoplasma	[119, 120]
Lipoteichoic acid	TLR2	Recognition of Gram (+) bacteria	[121]
Zymosan	TLR2	Recognition of fungi	[122]
Double-stranded RNA	TLR3	Recognition of virus	[123]
Single-stranded RNA	TLR7 and TLR8	Recognition of virus	[124, 125]
Flagellin	TLR5	Recognition of Gram (−) bacteria	[126]
Unmethylated CpG DNA	TLR9	Recognition of bacteria and virus	[127, 128]
*β*-amyloid	TLR2; TLR4; TLR4 and TLR6	Neuroinflammation in Alzheimer's disease	[95, 96, 129, 130]
Mitochondrial DNA	TLR9	Pathogenesis of myocarditis and heart failure	[128]
Lung surfactant protein-A and -D	TLR4 TLR2	Innate immune component of lung. Act as opsonin and macrophage activator. Physiological implications of excessive activation by TLR is not known	[131–133]
Tenascin-C	TLR4	Maintenance and pathogenesis of inflammation in rheumatoid arthritis	[134, 135]
Fibrinogen	TLR4	Present normally in serum and activation has been implicated in rheumatoid arthritis and atherosclerosis	[136, 137]
Oxidised low-density lipoprotein	TLR4	Pathogenesis of atherosclerosis	[95]
MicroRNA let-7	TLR7	Pathogenesis of neurodegeneration	[138]

## Endnotes

1. PAMPs are conserved sequences or structural fragments on pathogens (nonself) that are recognised by PRRs. Examples of PAMP include bacterial, viral, fungal, and parasitic-derived lipids (lipopolysaccharide, lipoteichoic acid), proteins (flagellin), carbohydrates (mannan, zymosan), and nucleic acids (dsRNA, CpG).

2. DAMPs are endogenous molecules released from damaged cells (altered self). Examples of DAMP include heat shock proteins, ATP, and uric acid.

3. Toxoplasmosis is caused by *Toxoplasma gondii*. Cats are the definitive hosts and humans being intermediate hosts of *T. gondii*. Infection to humans spreads with contamination of food and water by cat faeces as well as eating undercooked meat infected with the parasitic cyst. Clinically, swelling of lymph nodes may occur but, interestingly, toxoplasmosis is associated with psychiatric disorders like schizophrenia, bipolar disorder, anxiety, and personality disorders.

4. Sleeping sickness is also known as Human African trypanosomiasis. It is caused by *Trypanosoma brucei* and is transmitted by tsetse fly. Prevalence is mainly in West, Central, and East Africa. It is characterised by intermittent fever and CNS manifestations in late stages including tremors, encephalopathy, and sleep disturbances which is mainly daytime somnolence.

5. Cerebral malaria is encephalopathy caused by sequelae of *Plasmodium falciparum *infection. Neurological features include coma, seizures, and upper-motor neuron lesion features (muscle spasticity and rigidity).

6. Neurocysticercosis is an infection of the CNS caused by the tapeworm *Taenia solium*. Pig is the intermediate host while humans are the definitive hosts of *T. solium. *Most common clinical presentation is seizures (an important and leading cause for acquired epilepsy) and focal neurological signs depending on the site and localisation of the cysts.

7. ALS is also known as motor neurone disease and Lou Gehrig's disease. Majority of the cases are idiopathic with however a small percentage (5–10%) being familial. Mutations in genes *SOD1 *(codes for Superoxide dismutase 1, an antioxidant); *TARDBP* (codes for Transactive response DNA-binding protein 43, a nuclear protein); and *FUS *(codes for Fused in Sarcoma, another cellular protein) are involved in familial ALS. It is a fatal, progressive neurodegenerative disease characterised by muscle spasticity, wasting and fasciculations as well as dysphagia and dysarthria. Interestingly, ALS is associated with frontotemporal dementia and this lead to discovery of mutation in *C9ORF72* gene (abnormal nucleotide repeats) in familial and sporadic forms of ALS [317–319].

8. Hydatidiform mole is a gestational trophoblastic disease. Trophoblasts are precursors to placental cells. The products of conception will completely or partially comprise of grape-like vesicles or sacs (villous trophoblast). Most pregnancies are not viable with presenting symptom being vaginal bleeding. Early diagnosis can be established by ultrasonography (“snowstorm” appearance).

9. Muckle-Wells syndrome is an autosomal dominant disease characterised by the presence of intermittent fevers, rashes, sensorineural hearing loss, and amyloidosis. Mutation occurs in gene *CIAS1*.

10. Metabolic syndrome refers to a combination of hyperglycemia, obesity, dyslipidaemia, and hypertension.

11. MicroRNAs are 22 nucleotide RNAs that are noncoding and repress expression of mRNAs.

## Abbreviations

A*β*: Amyloid-*β*
AD: Alzheimer's disease
BIR: Baculovirus inhibitor of apoptosis protein repeat
C4BP: C4b-binding protein
CARD: Caspase activation and recruitment domain
CNS: Central nervous system
Crry: Complement receptor 1-related protein-y
DAMP: Damage-associated molecular pattern
DOCK8: Dedicator of cytokinesis 8
HSV: Herpes simplex virus
HD: Huntington's disease
IL: Interleukin
LPS: Lipopolysaccharide
MAPK: Mitogen-activated protein kinase
MBL: Mannan-binding lectin
MASP: MBL-associated serine protease
MyD88: Myeloid differentiating factor 88
NF-*κβ*: Nuclear factor-*κβ*
NOD: Nucleotide-binding and oligomerisation domain
NLR: NOD-like receptors
NM: Neuromelanin
PAMP: Pathogen-associated molecular pattern
PD: Parkinson's disease
PRR: Pattern-recognition receptor
PYD: Pyrin domain
RAGE: Receptor for advanced glycation endproducts
SR: Scavenger receptor
SR-BI: Class B SR type I
TBI: Traumatic brain injury
TLRs: Toll-like receptors
TNF: Tumour necrosis factor
WNV: West Nile virus.
